# A-Type Proanthocyanidins from the Stems of *Ephedra sinica* (Ephedraceae) and Their Antimicrobial Activities

**DOI:** 10.3390/molecules18055172

**Published:** 2013-05-06

**Authors:** Xinyu Zang, Mingying Shang, Feng Xu, Jing Liang, Xuan Wang, Masayuki Mikage, Shaoqing Cai

**Affiliations:** 1 State Key Laboratory of Natural and Biomimetic Drugs, School of Pharmaceutical Sciences, Peking University, Xueyuan Road, Beijing 100191, China; 2 Graduate School of Natural Science & Technology, Kanazawa University; Kakuma-machi, Kanazawa 441-1212, Japan

**Keywords:** *Ephedra sinica*, Ephedraceae, A-type proanthocyanidin, ephedrannin D_1_, ephedrannin Tr_1_, ephedrannin Te_1_, antimicrobial activities

## Abstract

Phytochemical investigation of the *n*-BuOH-soluble fraction of the EtOH extract of the herbaceous stems of *Ephedra sinica*, which is known as Ephedrae Herba in Traditional Chinese Medicine, led to the isolation and identification of 12 A-type proanthocyanidins, containing five dimers, two trimers and five tetramers [*i.e.*, (+)-epigallocatechin-(2*α*→*O*→7,4*α*→8)-(-)-catechin, named ephedrannin D_1_, a dimer; epigallocatechin-(2*α*→*O*→7,4*α*→8)-epigallocatechin-(4*α*→8)-catechin (ephedrannin Tr_1_), a trimer; and epigallocatechin-(2*α*→*O*→7,4*α*→8)-epigallocatechin-(4*α*→8)-epigallocatechin-(2*α*→*O*→7,4*α*→8)-gallocatechin, named ephedrannin Te_1_, a tetramer). Tetramers composed of gallocatechin are reported for the first time in Ephedraceae. Catechin, epicatechin, gallocatechin, epigallocatechin and four known dimers were also isolated. The structures were elucidated by extensive spectroscopic analysis. The absolute configurations of the 4*α* linkages, which were confirmed by NOESY and CD experiments, are the outstanding characteristic of most of these isolated A-type proanthocyanidins. The antimicrobial activities of these compounds were tested by measuring the minimum inhibitory concentrations (MIC) against bacteria (both Gram positive and Gram negative) and fungi, and were found to be in the range of 0.00515–1.38 mM. Compounds **6**, **8**, **10** and **11** exhibited moderate antimicrobial activities against *Canidia albicans*.

## 1. Introduction

*Ephedra sinica* Stapf. (Ephedraceae) known as Ephedrae Herba (“Mahuang” in Chinese), has been used as an important medicinal herb in Traditional Chinese Medicine for thousands of years, and it is famous for containing six alkaloids of the ephedrine series [(-)-ephedrine, (+)-pseudoephedrine, (-)-*N*-methylephedrine, (+)-*N*-methylpseudoephedrine, (-)-norephedrine, (+)-norpseudoephedrine] [1]. According to the Chinese Pharmacopeia [2] and Japanese Pharmacopeia [3], “Ephedra Herb” is derived from the dried herbaceous stems of *Ephedra sinica* Stapf., *E. intermedia* Schrenk et C. A. Mey. or *E. equisetina* Bge., and is used for the treatment of asthma and cough, and as a diaphoretic. For years, ephedrine alkaloids were considered to be the main pharmacoactive constituents and few non-alkaloid-constituents was reported.

Nowadays, there has been considerable research on the bioactivities of proanthocyanidins, including antibacterial, antiviral, anticarcinogenic, anti-inflammatory, antiallergic, and vasodilatory effects [4,5,6,7], and primarily their antioxidant activity. Tannins, mainly proanthocyanidins, were proved by colorimetric reactions to occur in large amounts in the stems of many species of *Ephedra* (e.g., Eurasian *Ephedra*: *E. intermedia*, *E. przewalskii*, *E. alata*, *E. distachya* and *E. fragilis*; North American species of *Ephedra*: *E. californica, E. fasciculata*, *E. nevadensis*, *E. torreyana*, *E. trifurca* and *E. viridis*) [8]. The Eurasian *Ephedra* species contain ephedrine alkaloids, but the North American species of *Ephedra*, known as “Mormon tea” is believed to not contain significant amounts of ephedrine alkaloids [8]. The phytochemical basis behind the purported stimulant and therapeutic nature (such uses include cough medicines, an antipyretic, an antisyphilitic, a stimulant for poor circulation, and an antihistamine [9]) of “Mormon tea” produced from North American *Ephedra* is thus likely a result of their proanthocyanidin content [8], and hence proanthocyanidins may also play an important role in Asian species of *Ephedra*. For example, the stem of *E. distachya*, contains condensed tannins (including proanthocyanidins) that decrease the effects of uremic toxicity after kidney failure in rats [10]. Ephedranin A and B, both belonging to the A-type proanthocyanidins and considered to possess anti-inflammatory [11] and cytotoxic effects [12], were isolated from the root of *E. sinica* (called Ephedrae Radix and used as an antiperspirant in Traditional Chinese Medicine). Therefore it is obvious that proanthocyanidins may also play an important role in the pharmacological actions of Ephedrae Herba. However, proanthocyanidins of the stem of Ephedrae Herb and their bioactivities remain unknown.

In this study, four monomers, nine dimers, two trimers and five tetramers of A-type proanthocyanidins were isolated from the *n*-BuOH-solutable fraction of the EtOH extract of the herbaceous stems of *E. sinica*, among which the structures of 12 unknown compounds were determined by extensive spectroscopic techniques.

## 2. Results and Discussion

### 2.1. Chemistry

From the *n*-BuOH-solutable fraction of the EtOH extract of the herbaceous stems of *E. sinica*, 12 A-type proanthocyanidins **1**–**12** which are new compounds and include five dimers, two trimers and five tetramers, were isolated and identified together with eight known compounds **13**–**20**. Tetramers composed of gallocatechin are reported for the first time in Ephedraceae.

**Figure 1:**
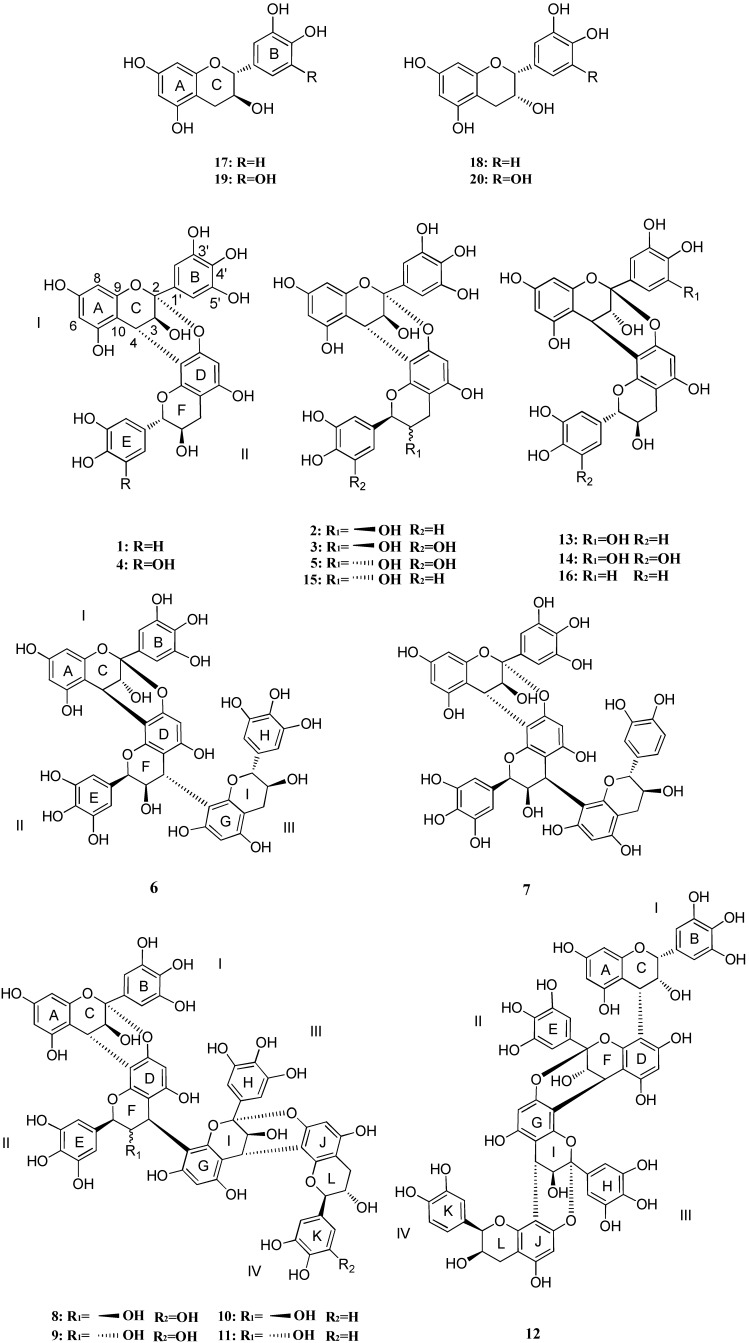
The chemical structures of compounds **1**–**20**.

For compounds **13** and **14**, which are mentioned in an earlier report [13], we provided for the first time their spectroscopic data, and named them ephedrannin D_2_ and ephedrannin D_5_. Compounds **15** and **16** were identified as (+)-epigallocatechin-(2*α*→*O*→7,4*α*→8)-(+)-catechin and (-)-epicatechin-(2*β*→*O*→7,4*β*→8)-(-)-catechin (proanthocyanidin A_4_) with reference to previous reports [14,15]. Compounds **17**–**20** were identified as catechin (**17**), epicatechin (**18**), gallocatechin (**19**), and epigallocatechin (**20**) by comparing their NMR spectroscopic data with authentic samples and literature data. The ^1^H- and ^13^C-NMR chemical shifts of the compounds **1**–**15** are summarized in Table 1, Table 2, Table 3, and their structures are depicted in Figure 1.

**Table 1:** ^1^H-NMR(400 MHz) spectroscopic data for **1**–**5** and **13**–**15** (in CD_3_OD, *δ* in ppm, *J* in Hz).

Unit	Position	1	2	3	4	5	13	14	15
І	3	4.10(3.6)	4.16(3.5)	4.16(3.5)	4.09(3.6)	4.14(3.6)	4.09(3.6)	4.07(3.5)	4.13(3.5)
	4	4.28(3.6)	4.41(3.5)	4.42(3.5)	4.28(3.6)	4.27(3.6)	4.29(3.6)	4.28(3.5)	4.25(3.5)
	6	6.05(2.2)	5.89(2.3)	5.90(2.3)	6.04(2.2)	5.94(2.3)	6.02(2.3)	6.02(2.3)	5.94(2.3)
	8	6.10(2.2)	6.07(2.3)	6.07(2.3)	6.09(2.2)	6.07(2.3)	6.09(2.3)	6.09(2.3)	6.07(2.3)
	2′	6.76 ^a^	6.75 ^a^	6.75 ^a^	6.76 ^a^	6.75 ^a^	6.77 ^a^	6.76 ^a^	6.75 ^a^
	6′	6.76 ^a^	6.75 ^a^	6.75 ^a^	6.76 ^a^	6.75 ^a^	6.77 ^a^	6.76 ^a^	6.75 ^a^
II	2	4.65(7.0)	5.04^c^	4.98 ^c^	4.67(6.1)	4.72(7.5)	4.65(6.9)	4.63(6.4)	4.75(7.9)
	3	4.00 ^b^	4.26 ^c^	4.24 ^c^	4.00 ^b^	4.05 ^b^	4.05 ^b^	4.05 ^b^	4.07 ^b^
	4	2.90(5.2, 16.4), 2.60(7.7, 16.4)	2.93(4.3, 17.0), 2.86(2.5, 17.0)	2.93(4.2, 17.0), 2.86(2.5, 17.0)	2.80(4.9, 16.4), 2.61(6.8, 16.5)	2.93(5.3, 16.4), 2.57(8.3, 16.3)	2.93(5.2, 16.6), 2.59(7.5, 16.5)	2.88(5.0, 16.6), 2.60(7.0, 16.6)	2.97(5.4, 16.3), 2.57(8.7, 16.3)
	6	6.09 ^a^	6.10 ^a^	6.10 ^a^	6.10 ^a^	6.09 ^a^	6.09 ^a^	6.10 ^a^	6.10 ^a^
	2′	6.80(2.0)	7.14(2.0)	6.67^a^	6.35^a^	6.54 ^a^	6.83(2.0)	6.38 ^a^	6.98(1.6)
	5′	6.76(8.2)	6.85(8.2)	-	-	-	6.78(8.2)	-	6.85(8.1)
	6′	6.70(8.2, 2.0)	6.96(8.2, 2.0)	6.67 ^a^	6.35 ^a^	6.54 ^a^	6.71(8.2, 2.0)	6.38 ^a^	6.88(1.6, 8.1)

^a^ singlet, ^b^ mutiplet, ^c^ broad singlet.

**Table 2:** ^1^H-NMR (400 MHz) spectroscopic data for **6**–**12** (in CD_3_OD, *δ* in ppm, *J* in Hz).

Unit	Position	6	7	8	9	10	11	12
І	2	-	-	-	-	-	-	5.47^c^
	3	4.17(3.3)	4.15(3.3)	4.19(3.5)	4.05(3.5)	4.19(3.5)	4.05(3.5)	4.23^c^
	4	4.31(3.3)	4.28(3.3)	4.48(3.5)	4.16(3.5)	4.47(3.5)	4.16(3.5)	4.90^c^
	6	5.87(2.3)	5.87(2.3)	5.93(2.3)	5.98(2.4)	5.92(2.3)	5.98(2.4)	5.91(2.4)
	8	6.00(2.3)	6.00(2.3)	6.07(2.3)	6.04(2.4)	6.07(2.3)	6.04(2.4)	6.06(2.4)
	2′	6.76 ^a^	6.75 ^a^	6.76 ^a^	6.71 ^a^	6.75 ^a^	6.71 ^a^	6.76 ^a^
	6′	6.76 ^a^	6.75 ^a^	6.76 ^a^	6.71 ^a^	6.75 ^a^	6.71 ^a^	6.76 ^a^
II	2	5.17 ^c^	5.16 ^c^	5.46 ^c^	4.62(9.9)	5.47 ^c^	4.62(9.9)	
	3	4.16 ^c^	4.16 ^c^	4.23 ^c^	4.80 ^d^	4.23 ^c^	4.80 ^d^	4.19(3.6)
	4	4.83 ^c^	4.82 ^c^	4.88 ^c^	4.75(7.6)	4.88 ^c^	4.75(7.6)	4.48(3.6)
	6	6.13 ^a^	6.13 ^a^	5.97 ^a^	5.84 ^a^	5.97 ^a^	5.83 ^a^	5.97 ^a^
	2′	6.51 ^a^	6.50 ^a^	6.65 ^a^	6.73 ^a^	6.65 ^a^	6.73 ^a^	6.65 ^a^
	5′	-	-	-	-	-	-	-
	6′	6.51 ^a^	6.50 ^a^	6.65 ^a^	6.73 ^a^	6.65 ^a^	6.73 ^a^	6.65 ^a^
III	2	4.73(7.5)	4.76(7.8)	-	-	-	-	-
	3	4.09 ^b^	4.10 ^b^	4.18(3.3)	4.16(3.4)	4.16(3.3)	4.15(3.5)	4.19(3.5)
	4	2.95(5.4, 16.4),2.59(8.3, 16.3)	2.99(5.5, 16.3), 2.59(8.8, 16.2)	4.29(3.3)	4.28(3.4)	4.26(3.3)	4.25(3.5)	4.42(3.5)
	6	5.91 ^a^	5.90 ^a^	5.81 ^a^	5.88 ^a^	5.80 ^a^	5.88 ^a^	5.76 ^a^
	2′	6.55 ^a^	6.98(1.8)	6.76 ^a^	6.79 ^a^	6.76 ^a^	6.79 ^a^	6.76 ^a^
	5′	-	6.88(8.2)	-	-	-	-	-
	6′	6.55 ^a^	6.55(8.2, 1.8)	6.76 ^a^	6.79 ^a^	6.76 ^a^	6.79 ^a^	6.76 ^a^
IV	2	-	-	4.70(7.5)	4.66(7.5)	4.73(7.5)	4.69(7.9)	5.01 ^c^
	3	-	-	4.07 ^b^	4.04 ^b^	4.09 ^b^	4.06 ^b^	4.25 ^c^
	4	-	-	2.93(5.4, 16.4), 2.56(8.2, 16.3)	2.93(5.4, 16.4), 2.54(8.4, 16.5)	2.98(5.4, 16.3), 2.56(8.7, 16.3)	2.98(5.6, 16.4), 2.54(8.8, 16.4)	2.93(4.3, 16.7),2.86(2.4, 17.2)
	6	-	-	6.11 ^a^	6.14 ^a^	6.12 ^a^	6.14 ^a^	6.12 ^a^
	2′	-	-	6.53 ^a^	6.52 ^a^	6.97(1.6)	6.96(1.6)	7.13(2.0)
	5′	-	-	-	-	6.84(8.2)	6.83(8.2)	6.84(8.2)
	6′	-	-	6.53 ^a^	6.52 ^a^	6.87(1.6, 8.2)	6.86(1.6, 8.2)	6.95(2.0, 8.2)

^a^ singlet, ^b^ mutiplet, ^c^ broad singlet.

**Table 3:** ^13^C-NMR(100 MHz) spectroscopic data for **1**–**15** (in CD_3_OD, *δ* in ppm).

Unit	position	1	2	3	4	5	6	7	8	9	10	11	12	13	14	15
I	2	100.6	100.5	100.5	100.6	100.5	100.6	100.6	100.5	100.4	100.5	100.4	78.6	100.6	100.5	100.5
	3	67.7	67.8	67.8	67.7	67.7	67.7	67.7	68.0	68.0	68.1	68.0	73.3	67.6	67.6	67.9
	4	29.7	29.2	29.2	29.7	29.2	29.4	29.4	29.3	29.5	29.5	29.6	36.2	29.5	29.5	29.3
	5	154.4	154.2	154.2	154.4	154.1	152.6	152.6	156.2	156.2	156.2	156.2	156.5	154.2	154.2	156.8
	6	97.0	98.0	98.0	97.09	98.2	96.5	96.5	98.3	98.6	98.4	98.7	98.3	97.7	97.7	98.2
	7	158.2	158.2	158.2	158.2	158.2	158.4	158.4	158.0	158.1	158.1	158.1	158.0	158.2	158.2	158.3
	8	96.6	96.6	96.6	96.6	96.6	95.7	95.8	96.6	96.7	96.6	96.7	96.6	96.6	96.6	96.7
	9	152.0	152.1	152.1	152.0	150.8	150.5	150.4	154.1	152.5	154.2	152.5	152.5	151.4	151.4	154.2
	10	104.3	104.1	104.1	104.3	104.1	102.1	102.1	105.3	104.6	105.3	104.6	104.6	104.4	104.4	104.2
	1′	131.4	131.6	131.5	131.6	131.5	132.1	131.9	132.0	131.6	132.0	131.6	131.4	131.5	131.4	131.5
	2′	107.7	107.6	107.6	107.7	107.6	107.4	107.4	107.4	107.7	107.5	107.7	107.6	107.7	107.7	107.7
	3′	146.4	146.4	146.4	146.4	146.4	146.3	146.3	146.3	146.4	146.4	146.4	146.4	146.4	146.3	146.5
	4′	134.7	134.7	134.7	134.7	134.7	134.8	134.8	134.6	131.6	134.7	134.67	134.7	134.7	134.7	134.7
	5′	146.4	146.4	146.4	146.4	146.4	146.3	146.3	146.3	146.4	146.4	146.4	146.4	146.4	146.3	146.5
	6′	107.7	107.6	107.6	107.7	107.6	107.3	107.3	107.4	107.7	107.5	107.7	107.6	107.7	107.7	107.7
II	2	82.8	80.9	80.9	82.6	84.0	77.2	77.2	78.6	85.4	78.6	85.4	100.5	82.6	82.5	84.0
	3	68.6	67.2	67.2	68.5	68.4	73.6	73.6	73.3	72.9	73.3	72.9	68.0	68.4	68.3	68.5
	4	28.3	29.5	29.4	27.4	28.4	36.4	36.4	36.2	40.0	36.2	40.1	29.3	28.0	27.4	29.0
	5	155.4	156.7	156.7	155.4	156.7	155.1	155.1	156.8	156.3	156.9	156.3	156.8	156.0	156.1	156.3
	6	96.5	96.5	96.5	96.4	96.5	96.5	96.5	96.3	99.4	96.6	99.3	96.3	97.6	97.6	96.6
	7	155.3	156.8	156.7	155.2	156.2	158.1	158.1	152.6	154.2	152.7	154.2	154.1	155.1	155.0	152.3
	8	108.7	107.0	106.9	108.6	106.6	102.6	102.7	104.6	103.2	104.6	103.1	105.3	108.6	108.6	106.6
	9	152.5	151.3	151.2	152.0	152.2	151.0	151.0	151.7	152.9	151.7	152.9	151.8	152.6	152.6	150.9
	10	103.5	101.9	101.9	103.3	102.7	106.9	106.9	106.2	110.1	106.3	110.2	106.3	101.8	101.6	102.9
	1′	132.2	131.5	130.7	131.5	130.4	132.0	130.9	131.3	130.0	131.4	130.0	131.1	132.1	131.5	131.1
	2′	115.0	115.3	106.9	106.7	107.4	107.0	107.0	107.0	108.9	107.1	108.9	107.0	115.0	106.8	115.5
	3′	146.3	146.3	146.7	146.9	147.1	146.7	146.7	147.1	147.2	147.1	147.2	147.0	146.3	146.9	146.5
	4′	146.3	146.3	134.1	133.9	134.6	133.5	133.5	133.9	134.7	134.0	134.7	133.9	146.2	134.0	146.9
	5′	116.1	116.2	146.7	146.9	147.1	146.7	146.7	147.1	147.2	147.1	147.2	147.0	116.2	146.9	116.4
	6′	119.8	119.4	106.9	106.7	107.4	107.0	107.0	107.0	108.9	107.1	108.9	107.0	119.7	106.8	120.4
III	2	-	-	-	-	-	84.0	84.0	100.5	100.7	100.5	100.7	100.5	-	-	-
	3	-	-	-	-	-	68.4	68.4	67.7	67.8	68.4	67.8	67.8	-	-	-
	4	-	-	-	-	-	28.4	28.9	29.4	29.3	29.3	29.3	29.4	-	-	-
	5	-	-	-	-	-	156.2	156.2	156.5	154.9	156.6	154.9	155.0	-	-	-
	6	-	-	-	-	-	99.4	99.3	99.2	98.0	99.2	98.0	99.0	-	-	-
	7	-	-	-	-	-	157.8	157.8	151.8	152.0	151.8	152.0	151.2	-	-	-
	8	-	-	-	-	-	104.0	103.9	103.6	109.8	103.6	109.8	103.6	-	-	-
	9	-	-	-	-	-	156.9	156.9	151.1	151.5	151.2	151.5	151.0	-	-	-
	10	-	-	-	-	-	109.5	109.4	109.4	106.7	109.4	106.7	109.3	-	-	-
	1′	-	-	-	-	-	130.3	129.9	131.7	131.9	131.8	131.9	132.1	-	-	-
	2′	-	-	-	-	-	107.4	115.2	107.6	107.5	107.7	107.5	107.6	-	-	-
	3′	-	-	-	-	-	147.1	146.5	146.4	146.4	146.4	146.4	146.3	-	-	-
	4′	-	-	-	-	-	134.5	146.4	134.7	135.1	134.7	135.1	134.7	-	-	-
	5′	-	-	-	-	-	147.1	120.4	146.4	146.4	146.4	146.4	146.3	-	-	-
	6′	-	-	-	-	-	107.4	116.3	107.6	107.5	107.7	107.5	107.6	-	-	-
IV	2	-	-	-	-	-	-	-	84.0	84.1	84.1	84.1	81.0	-	-	-
	3	-	-	-	-	-	-	-	68.4	68.5	67.7	68.5	67.2	-	-	-
	4	-	-	-	-	-	-	-	28.4	28.5	28.9	29.0	29.6	-	-	-
	5	-	-	-	-	-	-	-	155.0	156.0	155.0	156.0	156.6	-	-	-
	6	-	-	-	-	-	-	-	96.5	96.8	96.6	96.81	96.5	-	-	-
	7	-	-	-	-	-	-	-	156.9	156.7	156.8	156.8	156.8	-	-	-
	8	-	-	-	-	-	-	-	102.6	102.6	102.8	102.8	107.3	-	-	-
	9	-	-	-	-	-	-	-	150.5	150.6	150.6	150.7	151.8	-	-	-
	10	-	-	-	-	-	-	-	106.9	106.7	106.9	106.8	101.8	-	-	-
	1′	-	-	-	-	-	-	-	130.3	130.3	130.9	130.9	131.7	-	-	-
	2′	-	-	-	-	-	-	-	107.4	107.4	115.6	115.5	115.3	-	-	-
	3′	-	-	-	-	-	-	-	147.1	147.2	146.5	146.5	146.3	-	-	-
	4′	-	-	-	-	-	-	-	134.8	134.8	146.9	146.9	146.2	-	-	-
	5′	-	-	-	-	-	-	-	147.1	147.2	115.6	116.4	116.2	-	-	-
	6′	-	-	-	-	-	-	-	107.4	107.4	120.4	120.4	119.5	-	-	-

Compound **1**, an amorphous white powder, on TLC examination showed a typical reddish coloration characteristic of phenols with anisaldehyde-sulphuric acid reagent. The molecular formula was determined to be C_30_H_24_O_13_ by HR-ESI-TOF-MS, indicating **1** to be a dimeric proanthocyanidin. Its UV spectrum (HPLC-DAD) presented a band with maximum at 278 nm. All of the above data suggested that it belonged to the group of catechins/proanthocyanidins. The ^1^H-NMR spectrum showed an AX system for *δ*_H_ 4.10 (1H, d, *J* = 3.6 Hz, H-3) and 4.28 (1H, d, *J* = 3.6 Hz, H-4) in ring C, and the ^13^C-NMR spectrum showed a characteristic signal for a C-2 ketal carbon *δ*_C_ 100.6, suggested **1** to be an A-type of proanthocyanidin. In the aromatic area of the ^1^H-NMR spectrum, three singlets resonating at *δ*_H_ 6.80 (d, *J* = 2.0 Hz), 6.76 (d, *J* = 8.2 Hz) and 6.70 (dd, *J* = 2.0, 8.2 Hz), were assigned to the ABX system of a catechin moiety. The presence of a singlet at *δ*_H_ 6.76 integrating for two protons indicated the presence of a gallocatechin group. Further 2D NMR experiments (HSQC, HMBC, and NOESY) enabled the complete identification of the structure. The HMBC spectrum showed cross-peaks between the protons H-2′, 6′ (ring B) of the gallocatechin group and an oxygenated carbon at C-2 (*δ*_C_ 100.6), and between the H-4 (C ring) and C-2 (*δ*_C_ 100.6), which confirmed the presence of an epigallocatechin as the upper part (unit I) of compound **1**. Therefore, catechin was the terminal part (unit II) of the proanthocyanidin A-type skeleton. The 4→8 interflavanoid bond was confirmed by the key correlation between H-4 (ring C) and C-9 (ring D), H-2 (ring F) and C-9 (ring D). The NOESY experiment showed interactions between H-6 (ring D) and the aromatic protons H-2′, 6′ of ring B, and most importantly the cross-peak between H-3 (ring C) and H-6 (ring D). The latter one is considered to be of diagnostic importance, as it proves further the *trans*-stereochemistry of the 3,4-bond. The α-orientations at C-4 of the interflavan linkages were deduced from the diagnostic negative Cotton effect observed in the 220–240 nm region of the CD spectrum following the chiroptical rule which permits unambiguous assignment of absolute configuration at these chiral centers [16]. As the absolute configuration at position C-3 was characterized as 3*S* (β-hydroxyl group), based on the NMR spectroscopic data, the absolute configurations at positions 2, 3, 4 should be 2*R*, 3*S*, 4*S*, respectively. There is no distinguishing difference between compounds **15** and **1** in CD spectra (220–240 nm), but they differ in NMR data, especially in the unit II (Table 1, Table 3), which we can deduce that they are conformers with (+)-catechin or (-)-catechin as their terminal parts. As compound **15** was reported to have (+)-catechin as its terminal part, compound **1** was identified as (+)-epigallocatechin-(2*α*→*O*→7,4*α*→8)-(-)-catechin, a dimer, and named ephedrannin D_1_.

Compound **13**, an amorphous white powder, showed a reddish coloration with anisaldehyde-sulphuric acid reagent on TLC examination. The negative HR-ESI-TOF-MS of **13** showed a [M−H]^−^ peak at *m/z* 591.1136, which corresponded to a molecular formula of C_30_H_24_O_13_. The ^1^H-NMR and ^13^C-NMR spectra were similar to those of **1**, thus we concluded they were structural isomers. Further 2D NMR experiments (HSQC, HMBC, and NOESY) confirmed that **1** and **13** share the same relative configuration. The strong positive Cotton effect at 238 nm is consistent with the β-orientation of the C-4-flavan-3-ol groups [16], and the weak negative Cotton effect at 271 nm followed by a diagnostic positive effect at 287 nm was thought belonging to the 2α-phenyl (C ring)-2*α*-phenyl (F ring) structure [17]. On the basis of the relative configuration determination via NMR, the absolute configurations at positions 2, 3, 4 are designated as 2*S*, 3*R*, 4*R*, and compound **13** was identified as (-)-epigallocatechin-(2*β*→O→7,4*β*→8)-(-)-catechin, and named ephedrannin D_2_.

Compound **2**, an amorphous white powder, showed a reddish coloration with anisaldehyde-sulphuric acid reagent in TLC examination. Its molecular formula is C_30_H_24_O_13_, as deduced from HR-ESI-TOF-MS, showing the quasi-molecular ion [M−H]^−^ at *m/z* 591.1140. The ^1^H-NMR and ^13^C-NMR spectra were similar to those of **1**, except for the presence of signals [H-2 (*δ*_H_ 5.04) and H-3 (*δ*_H_ 4.26) (br.s)] in ring C, indicating an epicatechin unit. 2D NMR experiments confirmed that epicatechin was the terminal part of the proanthocyanidin A-type skeleton, and the 4→8 interflavanoid bonding. Based on this comparison and together with CD spectrum showing a strong (−)-CE at 238nm for the α-oriented C-2, 4 flavan-3-ol substituents, compound **2** was identified as (+)-epigallocatechin-(2*α*→*O*→7,4*α*→8)-(-)-epicatechin, and named ephedrannin D_3_.

Compounds **3**, **4**, **5** and **14**, amorphous white powders, on TLC examination showed typical reddish colorations characteristic of phenolics with anisaldehyde-sulphuric acid reagent. The HR-ESI-TOF-MS recorded in negative-ion mode exhibited deprotonated ions [M−H]^−^ at *m/z* 607.1086, 607.1088, 607.1082, and 607.1084, indicating C_30_H_24_O_12_ as their molecular formula. The ^1^H-NMR and ^13^C-NMR spectra suggested them to be dimeric A-type of proanthocyanidins composed of two gallocatechin groups. Comparisons have been made between the ^1^H-NMR, ^13^C-NMR, 2D NMR, and CD spectra of **2** and **3**. As **3** has an epigallocatechin group as the terminal part, it was identified as (+)-epigallocatechin-(2*α*→*O*→7,4*α*→8)-(-)-epigallocatechin, and named ephedrannin D_4_. At the same time, comparisons between **13** and **14** enabled the identification of the structure of **14** as (-)-epigallocatechin-(2*β*→*O*→7,4*β*→8)-(-)-gallocatechin, named ephedrannin D_5_. By comparison with **1**, compound **4** was identified as (+)-epigallocatechin-(2*α*→*O*→7,4*α*→8)-(-)-gallocatechin, named ephedrannin D_6_. By comparisons with compound **15**, (+)-epigallocatechin-(2*α*→*O*→7,4*α*→8)-(+)-catechin, we identified **5** to be (+)-epigallocatechin-(2*α*→*O*→7,4*α*→8)-(+)-gallocatechin, named ephedrannin D_7_.

Compound **6** was obtained as an amorphous white powder and showed a reddish coloration with anisaldehyde-sulphuric acid reagent on TLC examination. Its molecular formula was determined to be C_45_H_36_O_20_ by a HR-ESI-TOF-MS experiment, which suggested **6** to be a trimeric proanthocyanidin. The ^1^H-NMR spectrum of **6** revealed signals for three 3′,4′,5′-trisubstituted flavan-3-ol moieties, *i.e.*, three singlets at *δ*_H_ 6.51, 6.55, 6.76 each integrating for two protons indicated the presence of three gallocatechin groups and two singlets (*δ*_H_ 5.91 and 6.13) in the aromatic region. Two *meta*-coupled protons [H-6 (*δ*_H_ 5.87) and H-8 (*δ*_H_ 6.00) (*J* = 2.3 Hz)], and one AX system for [H-3 (*δ*_H_ 4.17) and H-4 (*δ*_H_ 4.31) (*J* = 3.3 Hz)] of the C-2 and C-4 doubly linked epigallocatechin residue. Two sets of signals characteristic for the H-2, H-3 and H-4 of a epigallocatechin residue [*δ*_H_ 5.17, H-2; *δ*_H_ 4.16, H-3; *δ*_H_ 4.83, H-4; ring F], and a gallocatechin residue [*δ*_H_ 4.73, d, *J* = 7.5 Hz; H-2; *δ*_H_ 4.09, m, H-3; *δ*_H_ 2.95, dd, *J* = 5.4, 16.4 Hz and 2.59, dd, *J* = 8.3, 16.3 Hz, H-4; ring I]. The HMBC spectrum showed cross-peaks between the protons H-2′, 6′ (ring B) of the gallocatechin group and the oxygenated carbon at *δ*_C_ 100.6 (C-2), and between H-4 of the C ring and *δ*_C_ 100.6 (C-2), which confirmed the presence of an epigallocatechin as the upper part (unit I) of compound **6**. From the ^1^H-NMR data, a gallocatechin group was deduced as the terminal part (unit III) from the presence of two H-4 protons (ring I). Thus, another epigallocatechin was assigned to be the middle unit (unit II) of compound **6**. The 4→8 interflavanoid bond was confirmed by the key HMBC correlations between H-4 (ring C) and C-9 (ring D), H-2 (ring F) and C-9 (ring D), H-4 (ring F) and C-9 (ring G), H-2 (ring I) and C-9 (ring G). The NOESY experiment also showed interactions between H-6 (ring D) and H-2′, 6′ (ring B), H-6 (ring G) and H-2′, 6′ (ring E). The CD spectra obtained for compound **6** was characterized by a weak Cotton effect at 275 nm and a strong positive Cotton effect at 238 nm. These bands are ascribed to the ^1^L_b_, ^1^L_a_ electronic transitions of the aromatic moieties in the flavan-3-ol rings. The Cotton effect at 238 nm is consistent with the *β*-orientation of the C-4 flavan-3-ol groups. On the basis of the relative configuration determination via NMR, together with correlation of the Cotton effect previously reported [16], compound **6** was established as epigallocatechin-(2*β*→*O*→7,4*β*→8)-epigallocatechin- (4*β*→8)-gallocatechin, a trimer, named ephedrannin Tr_1_.

The HR-ESI-TOF-MS data of **7** showed the [M−H]^−^ ion at *m/z* 895.1710, indicating a trimeric structure with C_45_H_36_O_19_ as its molecular formula. The differences in ^1^H-NMR and ^13^C-NMR data indicated that **6** and **7** have different terminal units. The characteristic signals of H-2, H-3, and H-4 of a catechin residue (*δ*_H_ 4.76, d, *J* = 7.8 Hz; H-2; *δ*_H_ 4.10, m, H-3; *δ*_H_ 2.99, dd, *J* = 5.5, 16.3 Hz and 2.59, dd, *J* = 8.8, 16.2 Hz, H-4; ring I) were detected in the ^1^H-NMR experiment. Additionally, the strong negative Cotton effect at 238 nm is consistent with the α-orientation of the C-4 flavan-3-ol groups. Compound **7** was thus identified as epigallocatechin-(2*α*→*O*→7,4*α*→8)-epigallocatechin-(4*α*→8)-catechin, named ephedrannin Tr_2_.

Compound **8** was obtained as an amorphous white powder and the molecular formula was determined to be C_60_H_46_O_28_ by HR-ESI-TOF-MS, indicating a tetrameric structure. Notably, the ^1^H-NMR spectrum displayed less complexity than anticipated, which may be attributed to the rigidity of the molecules associated with the presence of two doubly linked units in **8**, which was further confirmed by the presence of the two signals at *δ*_C_ 100.5 in the ^13^C-NMR spectra. The ^1^H-NMR spectrum of **8** revealed signals for four 3′,4′,5′-trisubstituted flavan-3-ol moieties, *i.e.*, four singlets at *δ*_H_ 6.53, 6.65, 6.76 and 6.76 each integrating for two protons indicated the presence of four gallocatechin groups, one AX system for two *meta*-coupled protons [H-6 (*δ*_H_ 5.93) and H-8 (*δ*_H_ 6.07) (*J* = 2.3 Hz)], and three singlets (*δ*_H_ 5.81, 5.97 and 6.11) in the aromatic region. Two AX system for [H-3 (*δ*_H_ 4.19) and H-4 (*δ*_H_ 4.48) (*J* = 3.5 Hz) (ring C)] and [H-3 (*δ*_H_ 4.18) and H-4 (*δ*_H_ 4.29) (*J* = 3.3 Hz) (ring I)] of the C-2 and C-4 doubly linked epigallocatechin residue. Two sets of signals characteristic for the H-2, H-3, and H-4 of a epigallocatechin residue (*δ*_H_ 5.46, H-2; *δ*_H_ 4.23, H-3; *δ*_H_ 4.88, H-4; ring F), and a gallocatechin residue (*δ*_H_ 4.70, H-2; *δ*_H_ 4.07, H-3; *δ*_H_ 2.93 and 2.56, H-4; ring L). In the HMBC spectrum, key correlations between H-4 (ring C) and C-9 (ring D), H-2 (ring F) and C-9 (ring D), H-4 (ring F) and C-9 (ring G), H-2 (ring F) and C-9 (ring G), H-4 (ring I) and C-9 (ring J), H-2 (ring L) and C-9 (ring J) were observed. The NOESY experiment also showed interactions between the H-6 (ring D) and H-2′, 6′ (ring B), H-6 (ring G) and H-2′, 6′ (ring E), H-6 (ring J) and H-2′, 6′ (ring H). The heterocyclic carbon signals of the upper (unit I and II) and terminal units (unit III and IV) of **8** were close to those of **3** and **5**, and the single signal at *δ*_H_ 4.88, H-4 of the ring F, together with the strong negative cotton effect at 238 nm in the CD spectra, established **8** as epigallocatechin-(2*α*→*O*→7,4*α*→8)-epigallocatechin-(4*α*→8)-epigallocatechin-(2*α*→*O*→7,4*α*→8)-gallocatechin, a tetramer, named ephedrannin Te_1_.

Compounds **8** and **9** differ in the ^1^H-NMR data at [*δ*_H_ 4.62 (*J* = 9.9 Hz) H-2; *δ*_H_ 4.80, H-3; *δ*_H_ 4.75, (*J* = 7.6 Hz), H-4] of ring F, indicating the presence of an gallocatechin residue. Thus, **9** was identified as epigallocatechin-(2*α*→*O*→7,4*α*→8)-gallocatechin-(4*α*→8)-epigallocatechin-(2*α*→*O*→7,4*α*→8)-gallocatechin, and named ephedrannin Te_2_.

Compounds **10** and **11** were obtained as amorphous white powders. The molecular formulae were determined to be C_60_H_46_O_27_ by HR-ESI-TOF-MS, indicating tetrameric structures. The ^1^H-NMR and ^13^C-NMR of **10** were quite similar to those of **8**, except for the singlets at *δ* 6.84 (d, *J* = 8.2 Hz), 6.87 (d, *J* = 1.6, 8.2 Hz), and 6.97 (d, *J* = 1.6 Hz) each integrating for one proton, that indicated the presence of one catechin group as its terminal unit (unit IV). Thus, **10** was identified as epigallocatechin-(2*α*→*O*→7,4*α*→8)-epigallocatechin-(4*α*→8)-epigallocatechin-(2*α*→*O*→7,4*α*→8)-catechin, named ephedrannin Te_3_. In the same way, by comparing its data with that of **9**, compound **11** was established as epigallocatechin-(2*α*→*O*→7,4*α*→8)-gallocatechin-(4*α*→8)-epigallocatechin-(2*α*→*O*→7,4*α*→8)-catechin, named ephedrannin Te_4_.

The HR-ESI-TOF-MS data of compound **12** showed the [M−H]^−^ ion at *m/z* 1197.2148, indicating a tetrameric structure with C_60_H_46_O_27_ as its molecular formula. The ^1^H-NMR spectrum of **12** revealed signals for three 3′,4′,5′-trisubstituted flavan-3-ol moieties and one 3′,4′-disubstituted flavan-3-ol moiety, *i.e.*, singlets at *δ*_H_ 6.65, 6.76, and 6.76 each integrating for two protons indicated the presence of three gallocatechin groups, one ABX system with singlets at *δ*_H_ 6.84, 6.95 and 7.13 indicating the presence of a catechin/epicatechin unit, one AX system for two *meta*-coupled protons [H-6 (*δ*_H_ 5.91) and H-8 (*δ*_H_ 6.06) (*J* = 2.4 Hz)], and three singlets (*δ*_H_ 5.76, 5.97 and 6.12) in the aromatic region, and two AX system for [H-3 (*δ*_H_ 4.19) and H-4 (*δ*_H_ 4.48) (*J* = 3.6 Hz) (ring F)] and [H-3 (*δ*_H_ 4.19) and H-4 (*δ*_H_ 4.42) (*J* = 3.5 Hz) (ring I)] of the C-2 and C-4 doubly linked epigallocatechin residue, two sets of signals having characteristics of the H-2, H-3, and H-4 of a epigallocatechin residue (*δ*_H_ 5.47, H-2; *δ*_H_ 4.23, H-3; *δ*_H_ 4.90, H-4; ring C), and a epicatechin residue (*δ*_H_ 5.01, H-2; *δ*_H_ 4.25, H-3; *δ*_H_ 2.93 and 2.86, H-4; ring L). The NOESY experiment showed clear interactions between the H-8 (ring A) and the aromatic protons H-2′, 6′ of ring B, H-6 (ring D) with H-2′, 6′ (ring B), H-6 (ring G) with H-2′, 6′ (ring E), and H-6 (ring J) with H-2′, 6′ (ring H). In the HMBC spectrum, correlations between H-2′, 6′ (ring H) and C-2 (ring I), H-2′, 6′ (ring E) and C-2 (ring F), H-2′, 6′ (ring B) and C-2 (ring C), confirmed the epigallocatechin-(4→8)-epigallocatechin-(2→*O*→7,4→8)-epigallocatechin-(2→*O*→7,4→8)-epicatechin linkages. Strong negative Cotton effect at 238 nm was detected in the CD spectra, which finally established compound **12** as epigallocatechin-(4*α*→8)-epigallocatechin-(2*α*→*O*→7,4*α*→8)-epigallocatechin-(2*α*→*O*→7,4*α*→8)-epicatechin, named ephedrannin Te_5_.

Literature research showed that only 13 trimers [18,19] and one tetramer [20] with A-type linkages composed of gallocatechin were reported. We reported two trimers and five tetramers of this kind. Furthermore, tetramers composed of gallocatechin are report for the first time in Ephedraceae. A-type proanthocyanidins with 4*α* linkages, the main type found in *E. sinica*, are less common in Nature than 4*β* ones, but in our work, 12 A-type proanthocyanidins with 4*α* linkages were isolated and identified.

### 2.2. Antimicrobial Activity

Antimicrobial activities of compounds **1**–**3**, **6**–**8**, **10**, **11**, **13** and **17**–**20** were determined by a serial dilution technique using 96-well microtiter plates [21]. The results are presented in Table 4 in terms of minimum inhibitory concentrations. Compound **11** showed the highest activity (MIC = 0.0835 mM) against the Gram-negative species *Pseudomonas aeruginosa*. Compounds **19** and **10** showed the highest activity (MIC = 0.0817, 0.0835 mM) against the Gram-positive species methicillin-resistant *Staphylococcus aureus*. Compound **8** were found to be the most active against fungi *Canidia albicans* (MIC = 0.00515 mM). In molar concentration terms, the order of activity against *C. albicans* was **8** > **10**, **11** > **6** > **3** >**7** > **1**, **2** > **13** > **19**.

All the tested compounds showed antibacterial and antifungal activities in different levels, which may, to some extent, correspond to the antimicrobial action [22] of Mahuang. Furthermore, compound **15**, previous isolated from *Quercus ilex* L. was reported to have antimicrobial activity (MIC = 0.17 mM) against *Pseudomonas aeruginosa* [14]. In our results, compound **1**, a conformer of compound **15**, possessed similar activity (MIC = 0.169 mM) against *Pseudomonas aeruginosa*.

**Table 4:** Minimum inhibitory concentrations MIC (mM) of the constituents of *E. sinica*.

Compd.	*Pseudomonas aeruginosa*	*Bacillus subtilis*	Methicillin-resistant *Staphylococcus aureus*	*Staphylococcus aureus*	*Escherichia coli*	*Canidia albicans*
1	0.169	0.676	0.676	0.338	>0.676	0.127
2	0.338	>0.658	0.338	>0.658	>0.658	0.127
3	0.338	0.658	0.338	0.658	>0.658	0.0626
6	0.439	>0.439	0.439	>0.439	>0.439	0.0274
7	0.112	0.446	0.223	0.446	>0.446	0.0838
8	0.334	>0.334	0.334	>0.334	>0.334	0.00515
10	>0.334	0.334	0.0835	0.334	>0.334	0.0104
11	0.0835	0.334	>0.334	>0.334	>0.334	0.0104
13	0.338	0.676	0.676	>0.676	>0.676	0.253
17	1.38	>1.38	>1.38	>1.38	>1.31	>1.38
18	0.653	>1.31	0.327	0.327	1.31	>1.31
19	1.31	1.31	0.0817	0.653	>1.31	0.653
20	0.345	>1.38	>1.38	0.172	0.653	>1.38
K	-	-	-	-	-	0.0000301
C	0.00302	-	-	-	0.00302	-
V	-	0.000354	0.000709	0.000709	-	-

K, ketoconazole; C, ciprofloxacin; V, vancomycin.

## 3. Experimental

### 3.1. General

Optical rotations were recorded on a JASCO DIP-140 digital polarimeter (Tokyo, Japan). IR spectra were measured on a Nicolet Nexus 470 infrared spectrometer (Madison, WI, USA). CD spectra were measured on a Jasco-810 CD spectrometer. NMR spectra were taken on Bruker AVANCE DRX 400 spectrometer (Fällanden, Switzerland), with tetramethylsilane (TMS) as an internal standard, and chemical shifts were indicated in *δ* values (ppm). HR-ESI-TOF-MS measurements were performed on a Waters Xevo G2 Q-TOF mass analyser (Milford, MA, USA). Column chromatography was performed with Amberlite XAD-2 gel (Sigma, Philadelphia, PA, USA) and Toyopearl HW-40C (TOSOH Corp., Tokyo, Japan). TLC was performed on silica gel GF_254_ (10–40 μm; Qingdao, China). Preparative HPLC was conducted on an Inertsil C18 column (20 mm i.d. × 250 mm, 5 μm) on a system equipped with a Shimadzu LC-20AP HPLC pump and a Shimadzu SPD-20A UV/VIS detector (Kyoto, Japan). All other chemical solvents used for isolation were of analytical grade (Beijing Beihua Fine Chemicals, Beijing, China and Wako Pure Chemical Industries, Osaka, Japan).

### 3.2. Plant Material

Dried herbaceous stems of *Ephedra Sinica* Stapf. were collected from Hangjin banner, Inner Mongolia, China, in May 2010, and the plant material was identified by one of the authors, Prof. Shao–Qing Cai. Its voucher specimen (No.6527) was deposited in the Herbarium of Pharmacognosy, School of Pharmaceutical Sciences, Peking University Health Science Centre (Beijing, China).

### 3.3. Extraction and Isolation

The dried and powdered herbaceous stems of *E. sinica* (35 kg) were sequentially extracted for 2 h each time under controlled reflux with EtOH-H_2_O (95:5, V/V, 3 × 280 L) and EtOH-H_2_O (1:1, V/V, 3 × 280 L). The combined extract solution was concentrated under reduced pressure to obtain a crude extract (5,880 g), and then the crude extract was suspended in H_2_O and successively partitioned with petroleum ether (60–90 °C), EtOAc, and *n*-BuOH.

**Figure 2:**
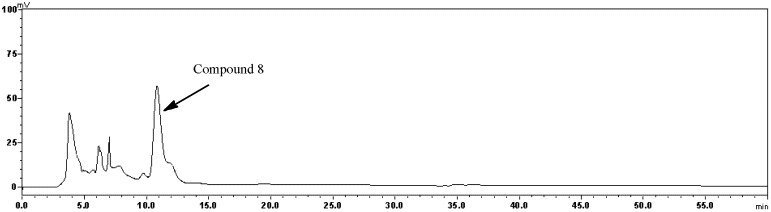
Preparative HPLC profile of compound **8 **(280 nm).

The *n*-BuOH-soluble part (300 g) was subjected to XAD-2 column chromatography (C.C.) and eluted with a H_2_O-MeOH gradient (1:0–0:1, v/v) to yield seven fractions (Fr.1–Fr.7). Fr.2 (15.0 g) was separated on a Toyopearl HW-40C column and eluted with a H_2_O-MeOH gradient (1:0–0:1, v/v) to afford five subfractions Fr.2A–Fr.2E. Compounds **5** (1.2 g) and **15** (1.5 g) were obtained from Fr.2B (42.0 g) and Fr.2C (55.2 g) by rechromatographed on Toyopearl HW-40C column and eluted with MeOH. Fr.2D (55.1 mg), Fr.2E (62.9 mg) and Fr.2F (31.1 mg) sub-eluates were rechromatographed in the same way to yield compounds **17** (15.1 mg), **18** (3.3 mg) and **19** (3.2 mg). Fr.3 (22.3 g) was applied to a Toyopearl HW-40C column and eluted with a H_2_O-MeOH gradient(1:0–0:1, v/v) to afford seven subfractions Fr.3A–Fr.3G. Fr.3D (57.3 mg) was rechromatographed on a Toyopearl HW-40C column and eluted with MeOH to yield compound **20** (2.1 mg). Compound **7** (15.0 mg) was obtained from the Fr.3G (62.9 mg) sub-eluate. Fr.4 (20.2 g) was applied to a Toyopearl HW-40C column and chromatographed in the same way. Compounds **2** (3.9 mg) and **14** (2.8 mg) were obtained from Fr.4A (12.7 mg) after rechromatography by preparative HPLC (5%–12%, 60 min, acetonitrile-water). Compounds **13** (3.8 mg) and **16** (2.1 mg) were isolated from Fr.4B (29.8 mg) by using the same conditions as for Fr.4A. Fr.4C (32.9 mg) was subjected to preparative HPLC using acetonitrile-water as mobile phase (5%–12%, 60 min) to yield compounds **1** (5.5 mg) and **4** (3.1 mg). Compounds **3** (2.3 mg) and **10** (16.0 mg) were obtained from the Fr.4D (36.9 mg) sub-eluate using the same conditions. Compounds **9** (4.2 mg) and **11** (3.1 mg) was obtained from Fr.4E (17.9 mg), while compounds **6** (5.3 mg) and **12** (2.1 mg) were isolated from the Fr.4F (28.6 mg) fraction. Fr.5 (30.9 g) was chromatographed on a Toyopearl HW-40C column with a H_2_O-MeOH gradient (3:2–0:1, v/v), and Fr.5B (26.9 mg) was subjected to preparative HPLC (5%–12%, 60 min, acetonitrile-water) to yield compound **8** (5.2 mg), Figure 2.

*Ephedrannin D_1_* (**1**). White amorphous powder (m.p. 252–254 °C (CHCl_3_)), 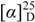 -8.1 (*c* = 0.10, MeOH); UV (MeOH) *λ*_max_ (log *ε*) 230, 278 nm; IR (film) *ν*_max_ 3422, 1638, 1393, 1343, 1167, 1055, 1032, 1013 cm^−1^; for ^1^H-NMR and ^13^C-NMR spectroscopic data, see Table 1, Table 3; HR-ESI-TOF-MS *m/z* 591.1134 ([M−H]^−^, calcd. for C_30_H_23_O_13_, 591.1139).

*Ephedrannin D_2_* (**13**). White amorphous powder (m.p. 245–248 °C (CHCl_3_)), 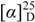 +16.0 (*c* = 0.10, MeOH); UV (MeOH) *λ*_max_ (log *ε*) 230, 278 nm; IR (film) *ν*_max_ 3452, 1637, 1346, 1054, 1032, 1009 cm^−1^; for ^1^H-NMR and ^13^C-NMR spectroscopic data, see Table 1, Table 3; HR-ESI-TOF-MS *m/z* 591.1136 ([M−H]^−^, calcd. for C_30_H_23_O_13_, 591.1139).

*Ephedrannin D_3_* (**2**). White amorphous powder (m.p. 256–258 °C (CHCl_3_)), 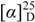 -9.3 (*c* = 0.10, MeOH); UV (MeOH) *λ*_max_ (log *ε*) 230, 278 nm; IR (film) *ν*_max_ 3433, 1620, 1450, 1344, 1142, 1084, 1037 cm^−1^; for ^1^H-NMR and ^13^C-NMR spectroscopic data, see Table 1, Table 3; HR-ESI-TOF-MS *m/z* 591.1140 ([M−H]^−^, calcd. for C_30_H_23_O_13_, 591.1139).

*Ephedrannin D_4_* (**3**). White amorphous powder (m.p. 231–233 °C (CHCl_3_)), 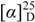 -10.2 (*c* = 0.10, MeOH); UV (MeOH) *λ*_max_ (log *ε*) 230, 278 nm; IR (film) *ν*_max_ 3734, 1624, 1444, 1103, 1019 cm^−1^; for ^1^H-NMR and ^13^C-NMR spectroscopic data, see Table 1, Table 3; HR-ESI-TOF-MS *m/z* 607.1086 ([M−H]^−^, calcd. for C_30_H_23_O_14_, 607.1088).

*Ephedrannin D_5_* (**14**). White amorphous powder (m.p. 244–245 °C (CHCl_3_)), 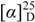 +23.9 (*c* = 0.10, MeOH); UV (MeOH) λ_max_ (log ε) 230, 278 nm; IR (film) *ν*_max_ 3625, 1631, 1382, 1058, 1034, 1015 cm^−1^; for ^1^H-NMR and ^13^C-NMR spectroscopic data, see Table 1, Table 3; HR-ESI-TOF-MS *m/z* 607.1084 ([M−H]^−^, calcd. for C_30_H_23_O_14_, 607.1088).

*Ephedrannin D_6_* (**4**). White amorphous powder (m.p. 250–252 °C (CHCl_3_)), 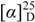 -16.0 (*c* = 0.10, MeOH); UV (MeOH) *λ*_max_ (log *ε*) 230, 278 nm; IR (film) *ν*_max_ 3432, 1628, 1341, 1179, 1142, 1011 cm^−1^; for ^1^H-NMR and ^13^C-NMR spectroscopic data, see Table 1, Table 3; HR-ESI-TOF-MS *m/z* 607.1088 ([M−H]^−^, calcd. for C_30_H_23_O_14_, 607.1088).

*Ephedrannin D_7_* (**5**). White amorphous powder (m.p. 233–234 °C (CHCl_3_)), 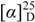 -21.4 (*c* = 0.10, MeOH); UV (MeOH) *λ*_max_ (log *ε*) 230, 278 nm; IR (film) *ν*_max_ 3447, 1634, 1341, 1179, 1112 cm^−1^; for ^1^H-NMR and ^13^C-NMR spectroscopic data, see Table 1, Table 3; HR-ESI-TOF-MS *m/z* 607.1082 ([M−H]^−^, calcd. for C_30_H_23_O_14_, 607.1088)

*Ephedrannin Tr_1_* (**6**). White amorphous powder (m.p. 217–218 °C (CHCl_3_)), 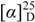 +89.0 (*c* = 0.10, MeOH); UV (MeOH) *λ*_max_ (log *ε*) 220, 270 nm; IR (film) *ν*_max_ 3447, 1634, 1456, 1166, 1107, 1017 cm^−1^; for ^1^H-NMR and ^13^C-NMR spectroscopic data, see Table 2, Table 3; HR-ESI-TOF-MS *m/z* 911.1678 ([M−H]^−^, calcd. for C_45_H_35_O_21_, 911.1671).

*Ephedrannin Tr_2_* (**7**). White amorphous powder (m.p. 203–205 °C (CHCl_3_)), 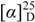 -91.6 (*c* = 0.10, MeOH); UV (MeOH) *λ*_max_ (log *ε*) 220, 270 nm; IR (film) *ν*_max_ 3448, 1629, 1450, 1147, 1107, 1055, 1017 cm^−1^; for ^1^H-NMR and ^13^C-NMR spectroscopic data, see Table 2, Table 3; HR-ESI-TOF-MS *m/z* 895.1710 ([M−H]^−^, calcd. for C_45_H_35_O_20_, 895.1722).

*Ephedrannin Te_1_* (**8**). White amorphous powder (m.p. 208–211 °C (CHCl_3_)), 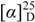 -130.3 (*c* = 0.10, MeOH); UV (MeOH) *λ*_max_ (log *ε*) 210, 270 nm; IR (film) *ν*_max_ 3485, 1632, 1445, 1350, 1112, 1055, 1011 cm^−1^; for ^1^H-NMR and ^13^C-NMR spectroscopic data, see Table 2, Table 3; HR-ESI-TOF-MS *m/z* 1213.2090 ([M−H]^−^, calcd. for C_60_H_45_O_28_, 1213.2097).

*Ephedrannin Te_2_* (**9**). White amorphous powder (m.p. 198–200 °C (CHCl_3_)), 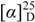 -162.9 (*c* = 0.10, MeOH); UV (MeOH) *λ*_max_ (log *ε*) 210, 270 nm; IR (film) *ν*_max_ 3715, 1618, 1444, 1366, 1100 cm^−1^; for ^1^H-NMR and ^13^C-NMR spectroscopic data, see Table 2, Table 3; HR-ESI-TOF-MS *m/z* 1213.2051 ([M−H]^−^, calcd. for C_60_H_45_O_28_, 1213.2097).

*Ephedrannin Te_3_* (**10**). White amorphous powder (m.p. 201–202 °C (CHCl_3_)), 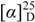 -136.1 (*c* = 0.10, MeOH); UV (MeOH) *λ*_max_ (log *ε*) 210, 270 nm; IR (film) *ν*_max_ 3424, 1626, 1450, 1350, 1177, 1142, 1033 cm^−1^; for ^1^H-NMR and ^13^C-NMR spectroscopic data, see Table 2, Table 3; HR-ESI-TOF-MS *m/z* 1197.2142 ([M−H]^−^, calcd. for C_60_H_45_O_27_, 1197.2148).

*Ephedrannin Te_4_* (**11**). White amorphous powder, (m.p. 203–205 °C (CHCl_3_)), 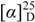 -89.3 (*c* = 0.10, MeOH); UV (MeOH) *λ*_max_ (log *ε*) 210, 270 nm; IR (film) *ν*_max_ 3424, 1626, 1450, 1351, 1169, 1142, 1033, 1011 cm^−1^; for ^1^H-NMR and ^13^C-NMR spectroscopic data, see Table 2, Table 3; HR-ESI-TOF-MS *m/z* 1197.2176 ([M−H]^−^, calcd. for C_60_H_45_O_27_, 1197.2148).

*Ephedrannin Te_5_* (**12**). White amorphous powder (m.p. 200–202 °C (CHCl_3_)), 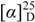 -110.2 (*c* = 0.10, MeOH); UV (MeOH) *λ*_max_ (log *ε*) 210, 270 nm; IR (film) *ν*_max_ 3445, 1631, 1506, 1350, 1114, 1065, 1007 cm^−1^; for ^1^H-NMR and ^13^C-NMR spectroscopic data, see Table 2, Table 3; HR-ESI-TOF-MS *m/z* 1197.2148 ([M−H]^−^, calcd. for C_60_H_45_O_27_, 1197.2148).

### 3.4. Antimicrobial Screening

Three Gram-positive bacteria (methicillin-resistant *Staphylococcus aureus*-clinical isolate), *Staphylococcus aureus* ATCC6538, *Bacillus subtilis* ATCC6633), two Gram-negative bacteria (*Escherichia coli* ATCC11229 and *Pseudomonas aeruginosa* PA01) and one fungi (*Canidia albicans* SC5314) were used as microorganisms in this assay.

Screening for *in vitro* anti-bacterial activity was performed according to the Antimicrobial Susceptibility Testing Standards outlined by the Clinical and Laboratory Standards Institute (CLSI, formerly NCCLS). The strains were recovered on LB agar plate overnight aerobically in 37 °C incubator, and adjusted to approximately 10^4^ CFU/mL with Mueller-Hinton Broth (Beijing AoBoXing Universeen Bio-Tech Co. Ltd., Beijing, China) as bacteria suspension. Aliquots (80 μL) of the diluted bacteria suspension were added to each well of the F-bottom 96-well sterile microplates (Greiner Bio-One Ltd., Frickenhausen, Germany), followed by the adding of 2 μL compound solutions in each test well. Two-fold serial dilutions of positive control drugs were added to the left column (column 1) on each 96-well plates as positive controls (positive control drugs used were vancomycin for *Bacillus subtilis*, *Staphylococcus aureus* and methicillin-resistant *Staphylococcus aureus* assay, ciprofloxacin for *Escherichia coli* and *Pseudomonas aeruginosa* assay, ketoconazole for the *Candida albicans* assay). Two μL of DMSO was added to each well of the right column (column 12) as negative control, which later showed no adverse effect on bacteria growth as compound solvent. After 16 h incubation at 37 °C aerobically, each well on 96-well plates was inspected for bacteria growth by OD_600nm_ measurement in PerkinElmer EnVision Multilabel Plate Reader (Waltham, MA, USA).

For MIC determination, overnight culture of the bacteria strains were diluted with fresh Mueller-Hinton Broth (Beijing AoBoXing Universeen Bio-Tech Co. Ltd.), and standardized to 2 × 10^4^ CFU/mL as bacteria suspension. Two μL of compounds solutions were added to row A of columns 2 to 11 on each 96-well plate containing 40 μL Mueller-Hinton Broth in each well, followed by a 2-fold serial dilution of each compound from row B to row H. Positive and negative controls were set up as described in the primary screening assay. Plates were incubated at 37 °C for 16 h and checked for bacteria growth. MIC here is defined as the lowest concentration of compound that results in inhibition of visible bacterial growth (no turbidity) compared with the positive control antibiotics.

## 4. Conclusions

Twelve new proanthocyanidins: (+)-epigallocatechin-(2*α*→*O*→7,4*α*→8)-(-)-catechin, named ephedrannin D_1_ (**1**), (+)-epigallocatechin-(2*α*→*O*→7,4*α*→8)-(-)-epicatechin, named ephedrannin D_3_ (**2**), (+)-epigallocatechin-(2*α*→*O*→7,4*α*→8)-(-)-epigallocatechin, named ephedrannin D_4_ (**3**), (+)-epigallocatechin-(2*α*→*O*→7,4*α*→8)-(-)-gallocatechin, named ephedrannin D_6_ (**4**), (+)-epigallocatechin-(2*α*→*O*→7,4*α*→8)-(+)-gallocatechin, named ephedrannin D_7_ (**5**), epigallocatechin-(2*β*→*O*→7,4*β*→8)-epigallocatechin-(4*β*→8)-gallocatechin, named ephedrannin Tr_1_ (**6**), epigallocatechin-(2*α*→*O*→7,4*α*→8)-epigallocatechin-(4*α*→8)-catechin, named ephedrannin Tr_2_ (**7**), epigallocatechin-(2*α*→*O*→7,4*α*→8)-epigallocatechin-(4*α*→8)-epigallocatechin-(2*α*→*O*→7,4*α*→8)-gallocatechin, named ephedrannin Te_1_ (**8**), epigallocatechin-(2*α*→*O*→7,4*α*→8)-gallocatechin-(4*α*→8)-epigallocatechin-(2*α*→*O*→7,4*α*→8)-gallocatechin, named ephedrannin Te_2_ (**9**), epigallocatechin-(2*α*→*O*→7,4*α*→8)-epigallocatechin-(4*α*→8)-epigallocatechin-(2*α*→*O*→7,4*α*→8)-catechin, named ephedrannin Te_3_ (**10**), epigallocatechin-(2*α*→*O*→7,4*α*→8)-gallocatechin-(4*α*→8)-epigallocatechin-(2*α*→*O*→7,4*α*→8)-catechin, named ephedrannin Te_4_ (**11**), and epigallocatechin-(4*α*→8)-epigallocatechin-(2*α*→*O*→7,4*α*→8)-epigallocatechin-(2*α*→*O*→7,4*α*→8)-epicatechin, named ephedrannin Te_5_ (**12**), were isolated, together with eight known compounds, from the stems of *E. sinica.* The antimicrobial activities of these compounds were tested by measuring the minimum inhibitory concentrations (MIC) against bacteria (both Gram positive and Gram negative) and fungi, which were found to be in the range of 0.00515–1.38 mM.
